# Measuring quality of life of old type 2 diabetic patients in primary care in Portugal: a cross-sectional study

**DOI:** 10.1186/2251-6581-13-68

**Published:** 2014-06-19

**Authors:** Filipe Prazeres, Daniela Figueiredo

**Affiliations:** 1 Faculdade de Medicina, Universidade de Coimbra, Polo III – Polo das Ciências da Saúde, 3000-548 Coimbra, Portugal; 2 Faculdade de Ciências da Saúde, Universidade da Beira Interior, 6200-506 Covilhã, Portugal; 3 School of Health Sciences, University of Aveiro, 3810-193 Aveiro, Portugal

**Keywords:** Elderly, Quality of life, Type 2 diabetes, Primary care

## Abstract

**Background:**

With the increasing prevalence of diabetes in patients aged over 75, the task of ensuring a good quality of life became even greater. This study aimed to evaluate quality of life of the very elderly (≥75 years) type 2 diabetic primary care patient, in an urban family practice setting.

**Methods:**

A cross sectional study was conducted. Quality of life (QoL) was assessed with the Portuguese version of EASY-Care. Descriptive and inferential analyses were performed.

**Results:**

Eighty three elderly type 2 diabetics were included in the study, with a mean age of 80.9 ± 4.3 years old. Most were women, widowed or married, with low educational levels, living with family members in urban areas and presented medium/low incomes. Participants were diagnosed with diabetes for 11.2 ± 10.1 years. Most of them were treated with oral antidiabetic agents, presented complications of diabetes and had good glycemic control. Despite that, excess weight, uncontrolled blood pressure and poor lipid management were noticed.

In general, the participants perceived a positive quality of life. The worst perceived domain was “mental health and well-being”. Within the sociodemographic variables, gender, monthly income, and living arrangements interfered the most with the quality of life domains. Studied clinical variables affected quality of life very modestly.

**Conclusions:**

In an urban primary care setting, when treating very elderly type 2 diabetic patients, and despite good glycemic control, attention should be paid to the QoL of women, the low income diabetics, their living arrangements and thoroughly evaluate the mental health and well-being of these patients.

## Background

During the 20^th^ century, and most probably the current one, aging was the most important demographic feature [1]. If in the year 2000 the elderly were represented by 600 million people, it is expected that in 2045 they will surpass the children, in number, worldwide [2], and 5 years later they will be a growing group of 2 billion [1].

There is an increased concern about the repercussions of our aging society in the social and health services worldwide but, at the same time, there is also the promotion of the idea that the elderly people need to take an active part in their quality of life through maintenance of mobility and independence [3].

Since aging has a multitude of patterns there isn’t a consensual definition of quality of life in this age group [4]. “Quality of life” in old age can be characterized as a complex, multidimensional, and holistic concept that includes social, environmental, structural, and health-related aspects [3].

The impact of aging rises in the old-old (≥75 years) with an increasing likelihood of chronic disease. For the next decade, chronic illnesses have been elected a priority by the WHO [5], in which Primary Care has an important role, providing comprehensive, person-centred care [6]. One of the biggest challenges of this century is warranting a good quality of life for the elderly, and this task is even greater when we deal with chronic diseases, particularly diabetes. Old age is an important risk factor for diabetes [7] and diabetes has been accepted as having a negative impact on quality of life [8]. Elderly diabetics show poor physical health and cognitive function in community health settings [9], particularly when associated with geriatric syndromes, hypoglycemia or diabetes complications [10], and when living in care homes they have reduced independence QoL scores [11].

With this study we aimed to examine the perceived quality of life (QoL) of older people with type 2 diabetes aged ≥75 years followed in an urban Family Health Unit and to analyse the associations between sociodemographic and clinical profiles and QoL. For these purposes, we used EASY-Care, which is an assessment instrument recommended by the WHO that establishes a link with quality of life concepts and the measurement of subjective wellbeing, enabling a multidimensional, comprehensive and systematic approach to quality of life in the primary care setting [12].

## Methods

### Study design and participants

A cross-sectional study was conducted between December 2009 and March 2010, in an urban Family Health Unit in the center region of Portugal. For the purpose of estimating sample size a diabetes prevalence of 26.3% [13] was used. We determined that a sample of 75 elderly patients (≥75 years old) was the minimum required, with a precision of 10% [14] and 95% CI, in accordance with 2001 Portuguese population census [15].

Participants were included if they: were ≥ 75 years old; had previous diagnosis of type 2 diabetes mellitus; had at least 1 appointment in the last 3 months; were able to sign the written informed consent and presented willingness and ability to comply with the study requirements. Participants were excluded if they: presented incapacity and/or inability to attend the interview; were diagnosed with dementia and/or presented inability to understand informed consent; or refused to participate.

The eligible participants were part of a population of 104 individuals aged ≥ 75 years, attending the diabetes clinical care of 6 family physicians. Each person within this population was contacted by telephone or mail address. A total of 83 older persons were included (80% response rate); 7 were excluded due to dementia diagnosis, 5 for being hospitalized, 1 refused to participate and 8 didn’t respond to telephone or letter invitation.

### Measures

Quality of life related data were collected using the EASY-Care (Elderly Assessment System), adapted to the Portuguese population [16]. This multidimensional instrument, available in 25 European Union countries, evaluates quality of life, well-being, social and health risks of the elderly people aged at least 75 years [16]. Includes the following domains: seeing, hearing and communicating; self-care; mobility; safety; accommodation and finance; staying healthy, mental health and well-being as well as medication administration. The answers to the questions of EASY-Care can be used to calculate three summary scores: i) independence score associated with need for care and support, which can vary between 0 and 100, with higher scores representing greater disability, ii) risk of breakdown in care, predicting the risk of hospital admission and whose score ranges between 0 and 12 points. High scores predict increased risk. iii) risk of falls, whose score ranges from 0 to 8. Three or more positive items indicate a high risk of falls. In addition, a score for each domain can be further calculated. High scores represent a worse perception of quality of life.

EASY-Care is recommended by the WHO to be used in primary and community care settings, to facilitate rapid and multidimensional assessment of older persons and prevent complications and fragilities. The reliability, validity and cost-effectiveness of this instrument have been tested worldwide with promising results [12,17].

Patient’s sociodemographics were collected by using the personal information section of EASY-Care.

Clinical data were collected from patients’ last appointment by consulting electronic or paper based medical records.

### Data collection

Data were collected in the context of a semi-structured interview. The same person (investigator) conducted all interviews in a private context, a medical office, or at the elderly’s household. The average time of the interview was approximately 1 hour. Data were treated in strict confidentiality to protect the privacy of patients. Each patient was assigned a consecutive number starting at 01.

### Data analysis

Data were processed using the statistical program PASW (Predictive Analytics SoftWare) Statistics 18. Descriptive and inferential analyses were performed. Parametric and nonparametric statistical tests were used for dependent (QoL) and independent variables (sociodemographic and clinical profiles). Mann–Whitney U Test was applied to compare the QoL (EASY-Care domains and final scores) between genders (except for the score “risk of breakdown in care” in which we used Independent Samples T-Test, as parametric requirements were fulfilled), living arrangements, duration of diabetes, glycemic control (except for the independence score in which we used Independent Samples T-Test, as parametric requirements were fulfilled). Kruskal-Wallis H Test was applied to compare the QoL (EASY-Care domains and final scores) between age groups (except for the score “risk of breakdown in care” in which we used One-Way ANOVA, as parametric requirements were fulfilled), marital status, scholarity, finances, BMI (except for the score “risk of breakdown in care” in which we used One-Way ANOVA, as parametric requirements were fulfilled), blood pressure, lipid profile, and diabetes-related complications.

A multiple regression was run to predict the summary scores of EASY-Care from the sociodemographic and clinical variables.

We considered statistically significant p values below 0.05.

### Ethical considerations

The study received full approval from the Ethics Committee of the Faculty of Medicine, University of Coimbra and by the Coordinator of the Family Health Unit, where the study took place. The study was conducted in accordance with the principles expressed in the Declaration of Helsinki. Written informed consents were obtained prior to any data collection. Older people who participated in this study were unpaid volunteers.

## Results

### Sample characterization

A total of 83 type 2 diabetic patients with a mean age of 80.9 ± 4.3 years were included in the study (Table 1).

**Table 1:** Study patients’ sociodemographic characteristics

	**n (%)**
**Gender**	
Male	29 (34.9)
Female	54 (65.1)
**Age (years)**	
75-79	37 (44.6)
80-84	26 (31.3)
85 and older	20 (24.1)
**Residence area**	
Rural area	6 (7.2)
Urban area	77 (92.8)
**Marital status**	
Married/cohabiting	36 (43.4)
Widowed	41 (49.4)
Single	3 (3.6)
Separated/divorced	3 (3.6)
**Living arrangements**	
Alone	27 (32.5)
With family members	56 (67.5)
**Formal education**	
Illiterate	28 (33.7)
1 to 4 years of education	46 (55.4)
5 or more years of education	9 (10.8)
**Professional status**	
Pensioner/retired	75 (90.4)
Housewife	8 (9.6)
**Monthly income**	
“Not enough to make ends meet”	37 (44.6)
“Just enough to make ends meet”	35 (42.2)
“Some money left over”	11 (13.3)

Table 2 shows the sample’s clinical variables. The mean (±SD) of self reported duration of diabetes was 11.2 ± 10.1 years. Mean glycosylated hemoglobin levels were 6.6 ± 1.0%. The average BMI of the sample was 29.3 ± 3.7 kg/m2. According to the WHO definition [18], 45.8% was clinically obese. Considering the BMI adjusted for the elderly population [19], 13.3% of the participants were malnourished or at risk of malnutrition. Diabetes-related complications were common (71.1%). High blood pressure was a frequent comorbidity (91.6%). The mean (±SD) systolic BP was 136 ± 19.9 mmHg and diastolic was 72 ± 10.0 mmHg. Mean (±SD) LDL levels were 98.3 ± 32.2 mg/dL, HDL were 49.0 ± 14.1 mg/dL and triglycerides were 113.2 ± 46.9 mg/dL.

**Table 2:** Study patients’ clinical characteristics

	**n (%)**
**Glycemic control**	
Excellent control (HbA1c < 7,0)	62 (74.7)
Good control (HbA1c 7,0-8,9)	16 (19.3)
Marginal control (HbA1c 9,0-9,9)	2 (2.4)
Bad control (HbA1c ≥ 10,0)	3 (3.6)
**Body Mass Index (kg/m**^**2**^**)**	
Normal (BMI 18,5-24,9)	11 (13.3)
Overweight (BMI 25–29,9)	34 (41.0)
Obese (BMI ≥ 30)	38 (45.8)
**Blood Pressure**	
Controlled^a^	23 (27.7)
Uncontrolled	60 (72.3)
**Lipid Profile**	
Controlled^b^	18 (21.7)
Uncontrolled	65 (78.3)
**Treatment**	
Diet and exercise only	1 (1.2)
Oral therapy only	74 (89.2)
Insulin only or combined	8 (9.6)
**Complications**	
No complications	24 (28.9)
Only microvascular^c^ complications	22 (26.5)
Only macrovascular^d^ complications	20 (24.1)
Microvascular and macrovascular complications	17 (20.5)
**Comorbidities**	
Hypertension	76 (91.6)
Osteoarthritis	37 (44.6)
Cardiac disease	29 (34.9)
Respiratory disease	20 (24.1)
Psychopathology	15 (18.1)
Malignant neoplastic disease	11 (13.3)

^a^BP < 130/80 mmHg.

^b^LDL <100 mg/dL, HDL >50 mg/dL and triglycerides <150 mg/dL.

^c^foot ulcer, blindness, photocoagulation or vitrectomy, dialysis, renal transplant, retinopathy, neuropathy and nephropathy.

^d^myocardial infarction, angina, heart failure, cardiac surgery, stroke, transient ischemic attack, and peripheral vascular disease.

### Perceived quality of life

Overall, participants perceived positively their quality of life in the considered domains. Nonetheless, it is noteworthy that the domain “mental health and well-being” had a mean score (9.8) which is very close to the midpoint of the theoretical range (2–18), making it the worst perceived domain (Table 3).

**Table 3:** EASY-Care domains (n = 83)

	**Mean ± SD**^**a**^	**Theoretical min.-max.**	**Observed min.-max.**
Seeing, hearing and communicating	1.6 ± 1.6	0-12	0-5
Self-care	9.8 ± 9.6	0-62	0-40
Mobility	3.9 ± 5.1	0-37	0-33
Safety	0.6 ± 0.7	0-5	0-3
Accommodation and finances	1.0 ± 1.6	0-5	0-5
Staying healthy	1.3 ± 0.7	0-5	0-3
Mental health and well-being	9.8 ± 3.4	2-18	2-16
Medication administration	0.5 ± 0.6	0-4	0-2

^a^Standard deviation.

It can be seen from the data in Table 4 that the studied sample had a low risk of falls, low risk of breakdown in care or hospital admission, and high independence (low need for support).

**Table 4:** Final summary scores of EASY-Care (n = 83)

	**Mean ± SD**^**a**^	**Theoretical min.-max.**	**Observed min.-max.**
Risk of falls	1.4 ± 1.2	0-8	0-5
Risk of breakdown in care or hospital admission	4.2 ± 2.2	0-12	0-9
Independence Score	14.0 ± 14.9	0-100	0-69

^a^Standard deviation.

### Analysis between sociodemographics, clinical variables and perceived quality of life

Women reported greater insecurity (p = 0.012) and had a worse perception of their mental health and well-being (p < 0.001) compared to men. Also the risk of breakdown in care or hospital admission was significantly higher in females (p = 0.003).

Participants with a high level of education had an “accommodation and finances” domain score that was significantly different from those that were illiterate (p = 0.036) and from those that had first grade or less (p = 0.044). The first group perceived better accommodation and finances.

Monthly income was significantly associated with five of the eight QoL domains (low-income patients reported worse QoL): (1) seeing, hearing and communicating (p = 0.002); (2) self-care (p = 0.002); (3) mobility (p = 0.048); (4) accommodation and finance (p = 0.010); (5) take medicines (p = 0.005). Low-income patients also presented an increased risk of falling (p = 0.042) and higher need for support (more dependent) (p = 0.001).

Older persons living with family members reported greater difficulty in self-care (p = 0.045) and in taking medicines (p = 0.018). Patients that lived alone manifested greater insecurity (p = 0.043) and worse mental health and well-being (p = 0.021) but greater independence (p = 0.025).

There were no significant differences between age groups and QoL and between marital status and QoL.

Participants with ≤10 years of type 2 diabetes onset reported greater insecurity (p = 0.016) but less difficulty in taking their medicines (p = 0.033).

Interestingly, for those subjects with good glycemic control (HbA1c <7%), greater difficulty in self-care (p = 0.037) was reported.

Well-nourished participants had a significantly higher mobility domain score, compared to those malnourished or at risk of malnutrition (p = 0.041) and to those that were obese (p = 0.013).

There were no significant differences between lipid profiles and QoL and between diabetes complications and QoL.

A multiple regression was run to predict the summary scores of EASY-Care from the sociodemographic and clinical variables. These variables only statistically significantly predicted the Independence Score, F(12, 70) = 3.755, p < 0.0005, adj. R2 = 0.29. Living arrangements, monthly income, and diabetes complications added statistically significantly to the prediction, p < .05.

## Discussion

The analysis of the responses to EASY-Care demonstrates that the majority of the very elderly type 2 diabetic patients had an overall positive quality of life. This finding is consistent with previous literature data, that shows maintenance of independence [20] and quality of life in older age [16] even in the context of chronic disease [21].

The present findings of a worse QoL regarding the domain mental health and well-being might be related to the association of chronic disease with depression [22] and loneliness [23] and the link of type 2 diabetes with cognitive decline in old age [24].

The results of the current study revealed more significant associations between the sociodemographic variables and QoL domains, compared to the clinical variables.

Gender, income and living arrangements interfered with QoL the most. The differences found in gender and income corroborate the results of a previous review, which suggested lower QoL scores for both females and low income diabetics [25]. In line with the previous literature [25,26] living alone had a negative effect on QoL (domains security and mental health and well-being). Subjects that did not live alone perceived greater difficulty in self-care and with the administration of medication. A possible explanation for this finding may be the fact that old diabetic patients experiment increasing functional impairment, which raises the need for support by a third-person, such as a family member or a caregiver.

In prior studies there is a considerable controversy regarding the relationship between duration of diabetes and quality of life [25]. The present findings demonstrated that there are no statistically significant differences in most domains of the EASY-Care among individuals with longer disease duration (over 10 years), compared to subjects who have diabetes for 10 years or less. These results can be explained by the observation that, in this study, the incidence of complications is independent of the duration of diabetes (chi-square (1) = 0.840, p = 0.359). However, these findings are not supported by the literature [27] in which patients suffering from chronic complications of diabetes have a longer duration of disease.

The literature is not concordant about the relationship between glycemic control and QoL [28]. The results of this study demonstrate that with exception of self-care, in which participants with good glycemic control reported greater difficulties in this domain, there are no statistically significant differences in other domains of EASY-Care. It seems likely that the deterioration in self-care may be due to the potential adverse effects of hypoglycemia, by maintaining HbA1c levels close to physiological values. Although HbA1c below 7% is recommended for the general diabetic population, this value has been questioned for the elderly diabetics and those with comorbidities [29,30]. It is advisable, therefore, that an individualized, less rigorous maintenance of low levels of HbA1c should be pursued in these patients [31,32].

There is a significant impairment of QoL in the obese [33,34] and also in the malnourished elderly [35]. This study confirms that the well-nourished elderly type 2 diabetic patient has better mobility.

In contrast to earlier findings [25,36,37], and except for the Independence Score, no evidence of association of diabetes complications with worse QoL was detected. Given the small sample size and the small number of elderly diabetics with chronic complications, further work is required to establish this with broader samples.

Extrapolation of the study results should be done with caution, regarding bias possibility: unicentral research study, univariate analysis as the main statistical approach, no control group was established, and a diabetes specific QoL questionnaire was not used. Despite these limitations, the use of EASY-Care, a generic instrument aimed to improve the care of the elderly, will allow future comparisons of the results of this study with other groups of elderly people with type 2 diabetes and also with different diseases. Will we find similar results in very elderly patients with other chronic diseases? Upcoming studies will decrease biases by being multicentral, with a defined control group, and by using both generic and specific QoL instruments.

## Conclusion

We believe that the findings of this study can make a contribution to daily practice. In an urban primary care setting, when treating very elderly type 2 diabetic patients, and despite good glycemic control, attention should be paid to their QoL, particularly of women and the low income diabetics, their living arrangements and thoroughly evaluate the mental health and well-being of these patients.

## Competing interests

The authors declare that they have no competing interests.

## Authors’ contributions

FP participated in the conception and design of the study, collected the data, performed the statistical analyses and drafted the manuscript. DF participated in the conception and design of the study, helped with the data analysis and with the draft of the manuscript. All authors gave their final approval of the version of the manuscript submitted for publication.
